# A study of ladder-like silk foothold for the locomotion of bagworms

**DOI:** 10.1038/s41598-021-95809-7

**Published:** 2021-08-17

**Authors:** Taiyo Yoshioka, Fumiko Yukuhiro, Tsunenori Kameda

**Affiliations:** Silk Materials Research Group, National Agriculture and Food Research Organization (NARO), 1-2 Owashi, Tsukuba, Ibaraki 305-8634 Japan

**Keywords:** Ecology, Evolution, Zoology

## Abstract

While walking on horizontal substrates, caterpillars skilfully engage all their legs, including three pairs of thoracic legs and a maximum of five pairs of prolegs, to move in a flexible wave-like motion. Such locomotory behaviours, represented by ‘crawling’ and ‘inching’ motions, have widely inspired the development of locomotion systems in soft robotics. However, bagworms are unable to use their prolegs for walking because these are always accommodated in a portable bag; thus, they are unable to walk using such general locomotory behaviours. Indeed, how they walk with only three pairs of thoracic legs is unknown at present. In this study, we show that bagworms construct a ladder-like foothold using their silk to walk without using prolegs. This enables them to walk not only on horizontal floor surfaces but also on wall and ceiling surfaces, even those with slippery or smooth surfaces. They construct the foothold by spinning a continuous silk thread in a zigzag manner and controlling the discharge of adhesive to attach the folded parts of the silk to a substrate. Discovery of this elaborate silk utilisation technique offers fresh insights into the diversity of silk use in lepidopteran larvae and provides potential designs for robot locomotion systems.

## Introduction

**Figure 1:**
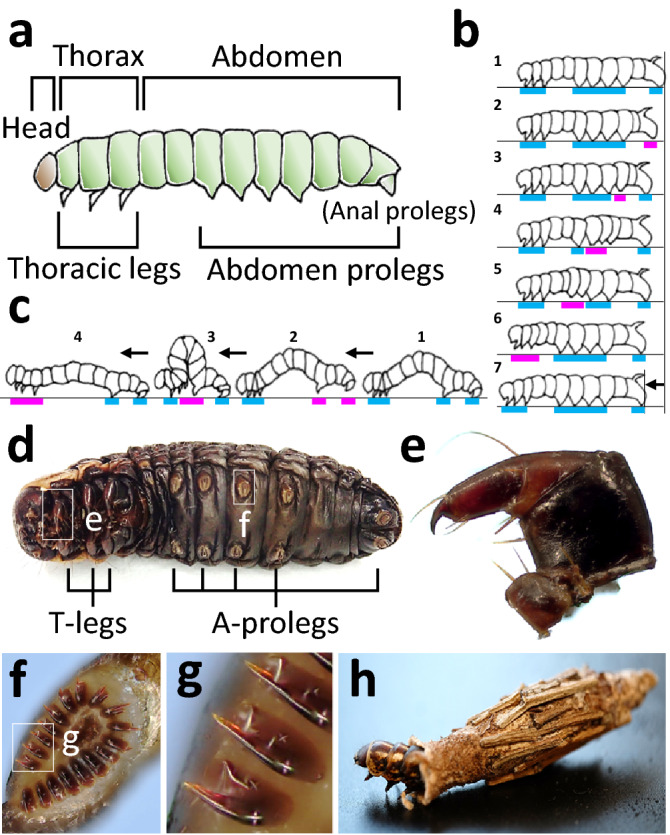
Caterpillar morphology. Schematic images of (**a**) the typical caterpillar morphology and (**b**,**c**) the representative caterpillar walking styles known as ‘crawling and ‘inching’. Images (**a**) and (**c**) are slightly modified from the original images by Sugiura et al.^2^ and (**b**) by Rogóż et al.^3^. These images are also inspired by a previous study^11^; as such, the segments of the caterpillar, which correspond to the legs and prolegs gripping the ground, are indicated using blue lines and those not in contact with the ground are shown using pink lines. (**d**) Ventral view of a final instar female larva of *Eumeta variegata*; enlarged images of this larva’s (**e**) thoracic legs and (**f**) abdomen prolegs [(**g**) shows a magnified image of three crockets from (**f**)]. (**h**) Image of a walking bagworm.

Lepidopteran larvae (i.e. caterpillars) generally have three pairs of thoracic legs on the thoracic segments, which are the true legs retained into adulthood, and a maximum of five pairs of prolegs on the abdominal segments (with an exception of up to seven pairs^1^), including a terminal pair on the hind-end referred to as anal (or caudal) prolegs (Fig. 1a^2^). When caterpillars walk on horizontal or gradually sloping substrates, they engage all legs skilfully to move in wave-like motions. The two main locomotory behaviours are ‘crawling’ and ‘inching’. The schematic images shown in Fig. 1b^3^ and c^2^ illustrate the sequence motion that occurs during a step in crawling (Fig. 1b: depicted from top to bottom) and inching (Fig. 1c: from right to left), respectively, in which the walking direction is depicted commonly from right to left. In both images, the segments, corresponding to the legs and prolegs gripping the ground, are indicated using blue lines and those not in contact with the ground are shown using pink lines. Inching is generally observed for small slender caterpillars, e.g. Geometridae caterpillars, which utilize their thoracic legs and prolegs as anchors alternatively in a step, during which a large portion of the mid-body loops upwards in an omega (Ω)-like shape (Fig. 1c). In contrast, crawling is observed for larger and thicker caterpillars, and the wave-like motion occurs segment-by-segment without the formation of the prominent loop observed in inching. In both locomotory behaviours, the prolegs play an inevitable role in providing the wave-like motions (detailed descriptions of crawling and inching are available in the literature^4–7^). Such caterpillar locomotion behaviours have served as an inspiration and foundational concept in robot locomotion systems. Particularly, replicating the stable, flexible and soft movements of caterpillars during crawling or inching, which facilitates a large carrying capacity^6,7^, is a goal of locomotion robotics, especially in soft robotics^3,8–11^. Notably, locomotion styles such as these are useless on vertical walls with slippery or smooth surfaces; nevertheless, caterpillars are sometimes found climbing such surfaces, e.g. the wall of a plastic cage. Roden^12^ found that the larvae of gypsy moths climb slippery walls by constructing a ladder-like silk foothold. They spin silk threads in a zigzag manner to produce a ladder-like shape (Roden termed this behaviour ‘laddering’), which they use to climb the slippery wall. To the best of our knowledge, however, the details of these ladder-like footholds, including their fine architecture, function and construction (i.e. spinning) mechanism have yet to be reported.

Bagworms are the larvae of bagworm moths (Lepidoptera: Psychidae), of which there are approximately 1000 species distributed worldwide^13^. Bagworms of all species commonly spin silk threads. Recently, we reported that the silk thread produced by Japan’s largest bagworm species, *Eumeta variegata*, is composed of a pair of thin filaments, each of which has an elliptical cross-section with an axial ratio of 1.7 in the major and minor axes, and is extraordinarily strong and tough in terms of its mechanical properties relative to those of other known natural silks^14^. Thus, bagworm silk is a promising candidate for the next generation of structural materials that will contribute to sustainable development goals. Bagworms are known to use their silk for at least four different purposes: nest building, dangling, ballooning and anchoring. First, the most well-known purpose is the construction of a portable nest, i.e. a ‘bag’. For this purpose, bagworms spin silk threads into a small nest (1–2 mm in length) immediately after hatching. In most cases, the exterior of the nest is covered with fine shavings of natural plant materials such as leaves and twigs^15,16^. The bag is made larger as the bagworm grows; in *E. variegata*, the bag reaches a length of 30–40 mm in males and 45–70 mm in females at the final instar stage. Second, for the purposes of dangling^2^, bagworms use their silk as lifeline or dragline from which they intentionally move down and up a tree or drop toward the ground as escape behaviour when disturbed. Third, for the purposes of ballooning, silk threads are released by early instars to catch the wind and allow lepidopteran larvae to disperse or migrate (this behaviour is also noted in spiders)^17^. Although ballooning can enable travel over long distances (e.g. > 100 km), bagworm larvae tend to use ballooning for short-distance dispersal within their original host tree rather than between trees^17,18^. Fourth, for the purposes of strongly anchoring their bag to leaves or twigs during moulting and pupation^19^, bagworms form a patch-like attachment disc consisting of looped and agglutinated silk threads (similar to spider attachment discs)^20^. Previously, Wolff et al.^19^ reported that the attachment disc of bagworms is composed of silk thread and independently discharged glue.

Typical of caterpillar morphology, bagworms have three pairs of thoracic legs and five pairs of prolegs including a pair of anal prolegs. For an example of this morphology in a final instar female of *E. variegata*, see the ventral view and magnified inserts in Fig. 1d–g. It is important to note that bagworms always carry their bag and that their prolegs are accommodated in the bag even during walking (Fig. 1h); hence, their prolegs are used to hold the bag from the inside via radially grown claws known as ‘crockets’ (Fig. 1f,g). This would suggest that bagworms may not be able to walk on slippery solid substrates, even those that are horizontal, when using only their three pairs of thoracic legs while carry their heavy bag.

In the present study, we address this potential locomotive difficulty by reporting a fifth purpose of silk in bagworms: it is used as a foothold to realise flexible locomotion (i.e. walking) not only on horizontal floor surfaces but also on the surfaces of walls and ceilings. Specifically, the bagworm spins a silk thread in a zigzag manner and constructs a ladder-like silk foothold by combining silk with an independently discharged adhesive. Here, we investigate the spinning mechanism that enables the construction of the foothold based on video-assisted observations of bagworm spinning behaviour and observations of morphologies from the silk gland to the spigot via optical microscopy for cross-sectioned samples and high-resolution 3D X-ray computer tomography (CT) for unsectioned samples.

## Results

### Bagworm walking method using a ladder-like silk foothold

**Figure 2:**
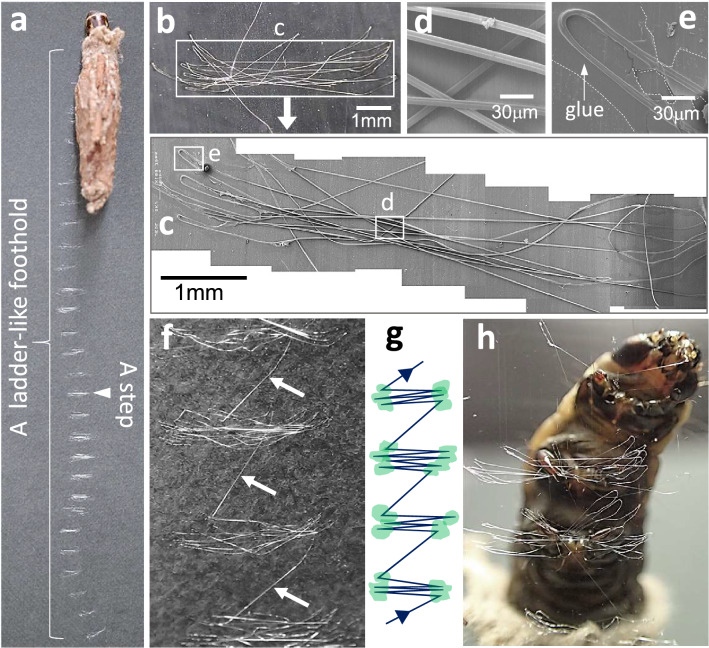
Architecture of the ladder-like foothold. (**a**) A typical ladder-like foothold constructed by a bagworm on black paper, (**b**) an enlarged image showing one of the steps in the foothold and (**c**) a scanning electron microscopy image of the step shown in (**b**). The unglued bridge threads and a glued turn in the step shown in (**c**) are magnified in (**d**) and (**e**), respectively. (**f**) An enlarged image of four continuous steps in the foothold shown in (**a**). The neighbouring steps are connected via a single thread indicated by the arrows. (**g**) A schematic depiction of the basic architecture of the foothold; blue lines and green circles correspond to the silk thread and glued parts, respectively. (**h**) A photograph of a bagworm constructing a foothold on a transparent plastic board.

When bagworms are reared in a plastic or glass cage, they walk not only on the floor but also on the walls or ceiling using only their three pairs of thoracic legs. The method by which they achieve this was clarified by placing a bagworm on black paper. Where the bagworm had walked, a ladder-like silk trace was observed on the black paper (Fig. 2a). Scanning electron microscopy (SEM) observation of one of the steps (or rungs) of the ladder-like trace revealed that each step was made up of a zigzag pattern of silk threads (Fig. 2b). Further magnified SEM observations revealed that the folded parts of the zigzag-spun thread were glued selectively to the substrate with adhesive whereas the remaining straight parts (hereafter, termed ‘bridges’ or ‘bridge threads’) were unglued (Fig. 2c–e).

Notably, the steps of the foothold were not independent but rather always connected with neighbouring steps via a single thread (Fig. 2f). The overall basic construction of the foothold is schematically depicted in Fig. 2g. We found that the foothold was constructed in one continuous movement and always made of a single thread regardless of walking distance or time; therefore, a continuous thread exceeding a length of 100 m could be collected from one foothold^14^. We also observed bagworm climbing behaviour on a transparent plastic board, which clarified the important role of the silk trace as a foothold (Fig. 2h). During this behaviour, the bagworm used its sickle claws (Fig. 1e) to hook its second and third pairs of thoracic legs onto the first and second newest steps, respectively, and constructed the next step by spinning silk with a zigzag motion of the head and the skilful use of the first pair of thoracic legs. When the bagworm advanced one step, it always first shifted its third pair of thoracic legs to the next step before then shifting its second pair of thoracic legs to the newest step to avoid overloading this step, which may not yet be fully adhered to the surface (see Supplementary Movie S1). Because of this construction method, the interval distance between neighbouring steps is automatically determined by the interval between the thoracic legs. By repeating this process, the bagworm can advance forward slowly but steadily. This walking method was commonly observed on a horizontal floor surface, vertical wall, or horizontal ceiling. Although we have mainly described and shown observations from *E. variegate* here, with the exception of Supplementary Fig. S4 and Movie S1, we also observed instances of walking behaviour in other species, namely *Eumeta minuscula*, *Mahasena aurea*, *Nipponopsyche fuscescens* and *Bambalina* sp. (for a movie on *E. minuscula* walking behaviour, wherein it climbs a vertical wall, see Supplementary Movie S2). For at least 100 individuals of these bagworm species, we observed essentially identical walking behaviour to that described in the present study without exceptions for locomotion on substrates with slippery surfaces.

**Figure 3:**
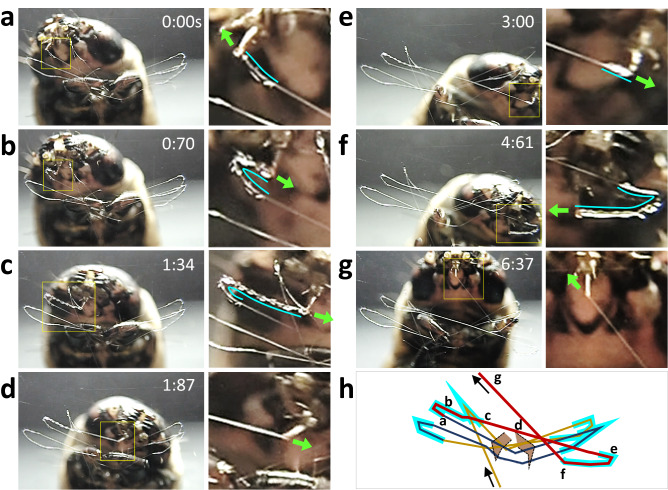
Foothold construction. (**a–g**) (left side) Time-sequence images taken during foothold construction and (right side) enlarged images of the vicinity of the spinneret (corresponding to the yellow rectangular area in each left-side image). The time-sequence images correspond to the parts of the schematic trace of foothold construction depicted by the red line in (**h**). In each right-side image and the schematic trace, the part of silk thread at which the adhesive was discharged is traced with a light-blue line. Green arrows in the right-side images show the direction of travel of the spinneret.

Based on our observations, we asked the following question: how do bagworms selectively glue the folded parts of the foothold onto the substrate? Real-time observation of the tip of the spinneret (i.e. the spigot) through a transparent plastic board during the construction of the foothold revealed that adhesive was selectively discharged to attach the folded parts to the substrate; this process could be distinguished from the continuous spinning of the silk thread (for a movie showing construction behaviour, see Supplementary Movie S3). Figure 3a–g shows a time-sequence of foothold construction with enlarged images in the vicinity of the spinneret provided, whereas Fig. 3h depicts a schematic trace of the construction process. It was clearly noted that the bagworm discharged the adhesive only at the folded parts (shown in Fig. 3a–c,e,f; termed the ‘glued turn’) and not at the straight bridge parts (shown in Fig. 3d,g; termed the ‘unglued bridge thread’). From these time-sequence observations, we concluded that the bagworm controls the discharge of adhesive in an ‘on and off’ manner as necessary (essentially the same construction behaviours were confirmed for at least 20 individuals).

### Passages of fibroin brins and adhesive

**Figure 4:**
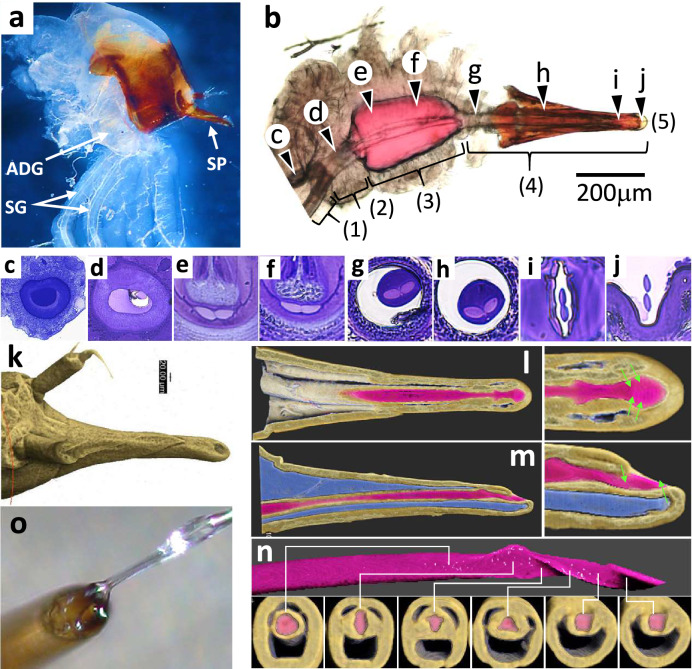
Structural examination of the passages of fibroin brins and adhesive. (**a**) An optical microscope image of the area in the vicinity of a spinneret isolated from a female bagworm in the final instar stage. Indicated by arrows is a pair of silk glands (SG), one of the adhesive glands (ADG) and the spinneret (SP). (**b**) An optical microscope image of the passage including the (1) end of the anterior SGs (ASGs), (2) common tube, (3) silk press, (4) spinning tube and (5) spigot. (**c–j**) Optical microscope images showing cross-sections of the passage of fibroin brins obtained from the corresponding positions (**c**–**j**) in image (**b**). To focus on the fibroin brins and its passage, the surrounding outer part was removed so that a pair of fibroin brins was revealed in each image (except for image (**c**), which shows only one side of the ASG). Unmagnified images of (**f**–**j**), including the outer part, are shown in Supplementary Fig. S2. (**k–n**) 3D X-ray CT images of the spinneret: (**k**) overview, (**l**) cross-sectional top view, (**m**) cross-sectional side view and (**n**) passage of the fibroin brins and corresponding cross-sectional images at various positions. In the cross-sectional side view (**m**), the sheath and core parts are coloured blue and pink, respectively. (**o**) Image of the tip of a spigot from which adhesive is overflowing and a silk thread is emerging.

We next investigated the spinning mechanism that enables continuous spinning of silk thread together with the selective discharge of adhesive via a single spigot. To this end, we observed the morphology of the bagworm from the silk gland to the spigot. Figure 4a shows the area in the vicinity of the spinneret, dissected and isolated from an *E. variegata* bagworm, which included a pair of silk glands and plural adhesive glands. As we previously reported^21^, the exterior shape of the silk gland in *E. variegata* (see Supplementary Fig. S1) is almost the same shape as that in the silkworm *Bombyx mori* and it is subdivided into three parts: the anterior (ASG), middle (MSG) and posterior (PSG) silk glands. We also previously confirmed that *fibroin heavy chain* (*h-fib*), *fibroin light chain* (*l-fib*) and *fiboinhexamerin* genes are expressed dominantly in the PSG, while *sericin* is expressed in the MSG, which strongly suggests that division-selective production of each protein exists in *E. variegata* (as has been shown in *B. mori*^22^). Figure 4b shows a magnified image of the spinneret including the end of the ASG. Beyond the pair of ASGs, which are merged into a common tube, a silk press and spinning tube appear before the spigot. This basic passage of silk fibroin from the ASG to the spigot is essentially the same as the passage observed in *B. mori*^23^. However, more detailed morphological observations of the inner structure of the passage revealed several obvious differences between *E. variegata* and *B. mori*.

Cross-sectional images along the spinneret are shown in Fig. 4c–j; these focus on the silk brins and their passage (unmagnified versions of the images in Fig. 4f–j are shown in Supplementary Fig. S2). The fibroin brins have an approximately round cross-sectional shape at the end of the ASG (Fig. 4c) and are merged at a common tube, which deforms their round shape slightly (Fig. 4d). The fibroin brins seem to be coated with a thin layer of sericin after the MSG, similar to *B. mori*; however, we omit the presence of the sericin layer here for convenience. The paired brins are gradually pressed between the ventral and dorsal hard cuticle plates at the silk press, and a gradual diameter decrease and shape deformation follows (Fig. 4e,f). At the exit of the silk press, each brin becomes elliptic and the diameter in the major axis decreases. Interestingly, the elliptical shape and 1.7-axial ratio for the major and minor axes of the fibroin brin cross-section in bagworm silk, which we previously reported^14^, are already determined at this stage in the silk press; afterwards, the diameter decreases without any change in the axial ratio of the elliptical cross-section. Notably, the two elliptical fibroin brins are aligned side-by-side so that their major axes are in line horizontally (to resemble a figure of ‘∞’) at the spinning press, and these are followed by the spinning tube (Fig. 4e–h). However, the alignment is twisted by 90° in one direction (to resemble a figure of ‘8’) before the brins are spun from the spigot (Fig. 4i,j).

We found that the spinning tube was surrounded by a hard exoskeleton. Using 3D-X-ray CT observations, we produced clear images of the exterior and interior morphologies of the spinning tube enveloped by exoskeleton (Fig. 4k–m; the exterior shape observed from the dorsal-, ventral- and lateral-sides by optical microscopy is provided in Supplementary Fig. S3). The spigot was not cut perpendicularly to the spinning tube but rather with a slope of around 20°; consequently, it was elliptic. X-ray CT clearly showed the core-sheath structure of the spinneret and a wide expanse of sheath parts (Fig. 4m) between the exterior shell and interior spinning tube (Fig. 4l,m). Using optical microscope observations of the cross-sections, we found that at least three pairs of adhesive ducts were running in the sheath space (Supplementary Fig. S2E). Therefore, while the silk brins pass through the central narrow spinning tube, the plural adhesive ducts pass through the outer space independently of the silk thread. Finally, the adhesive enters a ladle-like reservoir located at the spigot and is released together with the silk thread (Fig. 4o). The presence of definitive routes connecting the adhesive passage and the spigot were not clearly observed in our X-ray CT images, probably due to the small structural scale relative to the space resolution used in our analysis (i.e. 0.31 μm). We speculate that the adhesive merges into the spigot via a fine, porous sponge-like structure, and we indicate assumed routes in Fig. 4l,m. X-ray CT observations also revealed a sophisticated structural design involving gradual twists in the silk brins by 90° from ‘∞’ to ‘8’ (Fig. 4n and Supplementary Movie S4). Essentially identical spinneret structures were observed by X-ray CT images for all of eight observed individuals from the third to final instars of *E. variegata.*

## Discussion

In addition to the well-known uses of silk by bagworms, i.e. ballooning, dangling, nest production and anchoring, we have now described a fifth utilisation: walking. Specifically, we have shown that bagworms construct a ladder-like foothold by spinning a silk thread in a zigzag manner and selectively attaching the folded parts to a substrate using adhesive. By this method, the bagworm can walk in most situations (e.g. on a branch, leaf or tree trunk with a slippery surface or on the underside of a branch or leaf; Supplementary Fig. S4), even on artificially smooth materials, using only its three pairs of thoracic legs. However, the inevitable disadvantage of this method is the remarkably slow walking speed compared with the speed of general walking behaviours in caterpillars, including crawling and inching. Such a disadvantage would prove fatal if a caterpillar encountered a predator; however, bagworms can also quickly hide from predators in their portable protective bag, which they carry with them at all times. Thus, the foothold walking behaviour of bagworms may have evolved as a compromise to living with their protective bag. In actuality, bagworm larvae have lost the ability to walk using their prolegs; therefore, naked bagworm larvae, i.e., those without their bag, do not walk at all using their prolegs for crawling or inching behaviours, despite the fact that all their prolegs are free.

We have shown that the foothold construction process is surprisingly elaborate. The bagworm accurately controls adhesive discharge in an ‘on and off’ manner to facilitate rapid and selective gluing of folded parts in a zigzag-spun thread. Morphological observations of the spinneret via 3D-X-ray CT and optical microscopy clarified the spinning mechanism, i.e. independent discharge of the silk thread and the adhesive from a single spigot. Both the silk thread and adhesive pass through different pathways in the spinneret, which has a core-sheath structure, and merge at a ladle-like reservoir located at the tip of the spinneret. The ladle-like reservoir has a wide and elliptical exit cut at around 20°, which is a structure enabling the simultaneous discharge of a large amount of adhesive (Fig. 4o). This ladle-like reservoir may also play other important roles, e.g. in mixing plural adhesive components to activate adhesiveness.

The foothold construction behaviour of the bagworm, including the intentional ‘on and off’ adhesive discharge, is clearly different from the cocooning behaviour of *B. mori* and other lepidopteran species, in which the attachment of the silk thread on the inner surface of the cocoon during construction is accomplished only by the adhesiveness of sericin protein, which uniformly coats a pair of fibroin brins. Coating with sericin protein is not intentional but rather part of a self-organising protein system that occurs during the fibre formation process. Although the sericin layer in bagworms is much thinner than that observed in *B. mori* silk, the sericin protein of bagworms still plays an important role in merging two fibroin brins into a single thread and in attaching the thread to the inner surface of the bag during construction. It would be interesting to compare the foothold construction behaviour of the bagworm with that of gypsy moth larvae, which were observed first and possibly solely by Roden^12^. In his report, however, details about the fine architecture, construction behaviour (i.e. how the silk thread attaches to the substrate) and spinning mechanism were not provided. Therefore, a comparison of bagworm and gypsy moth caterpillar construction behaviours will be required in a future research project. In addition, in some species of cocooning larvae, inorganic-based secretions are discharged at the final stage of cocooning to strengthen the cocoon or reduce its porosity, e.g. the discharge of calcium oxalate in *Gonometa postica* or *Antheraea assama*^24,25^ (it must be emphasized that the glue of caterpillar silks is not limited to sericin only). Because in such cases a gland other than the silk gland produces the secretion, the behaviour may be similar to that of bagworms, even though the adhesive of the bagworm is not inorganic-based (further details on the adhesive components will be reported elsewhere). In future research, expression analysis of these adhesives could contribute to understanding lepidopteran evolution when combined with knowledge of silk gland types, silk fibroin molecular and crystalline structures and the phylogenetic relationships among silk-producing species^26–29^.

The elliptic cross-sectional shape of the fibroin brins, moulded at the silk press, guarantees a large contact area for attachment to the substrate. Indeed, the elliptic cross-sectional shape is rather flat with a high major-to-minor axes ratio of 1.7; thus, there is stable contact with the glued parts (Fig. 2e). Furthermore, the relationship between the geometries of the spigot and silk thread are practical for foothold construction. A pair of fibroin brins exits as a silk thread from the spigot where the major axes of the elliptic brins are approximately vertical. This figure of ‘8’ geometry provides efficient contact between the fibroin brins and the substrate with a minimum zigzag motion of the head, i.e. when the spigot is moved left and right by head shaking and is in contact with the substrate, the major axes of the elliptic brins are automatically parallel to the substrate (Fig. 3e). Our 3D-X-ray CT analysis reveals the surprisingly elaborate inner structure of the spinning tube, which enables the aligning direction of the elliptical brins to be twisted vertically (i.e. into a figure of ‘8’) slightly before the spigot.

In the present study, we have described and analysed the highly sophisticated utilisation of silk by bagworms for the purpose of walking for the first time. In summary, to compensate for not being able to use their prolegs for walking because they carry a portable bag for protection, bagworms construct ladder-like silk footholds that enable them to walk in any situation using only their three pairs of thoracic legs. The major difference between this foothold construction and the construction of cocoons by many other lepidopteran larvae is that bagworms can intentionally control the discharge of adhesive in an ‘on and off’ manner independent of their continuous spinning of silk thread. Consequently, they fabricate a highly elaborate silk architecture in which the glued and unglued parts are intentionally and accurately distinguished. Overall, this walking method is realised in bagworms through a combination of indispensable features, including the unique development of adhesive glands, the distinctive spinning behaviour and mechanism, the properties of the adhesive and silk thread and the elaborate design of the foothold architecture. In future studies, detailed comparisons of these features in bagworms and other lepidopteran larvae could provide fresh insights into the diversity of silk utilisation for survival and contribute to understanding the evolution of Lepidoptera. Furthermore, discovery of this sophisticated locomotive system paves the way for new concepts and designs in robot locomotive systems, especially those involving climbing walls or crawling on ceilings.

## Methods

### Observation of bagworms

*Eumeta variegata* bagworm larvae of fifth to final instars were collected in Tsukuba City, Ibaraki, Japan, during August to October in 2015 and 2019. Several body parts of final instar *E. variegata* bagworm larvae, such as the thoracic legs, prolegs and spinneret, were observed using an Olympus BX53 biological microscope equipped with a DP74 CMOS camera system (Olympus Co., Japan).

### Observation of silk footholds

Silk footholds constructed on various substrates were observed using a Stylus Tough digital camera (Olympus Co.) in microscope mode or a scanning electron microscope (JSM-6301F; JEOL Ltd., Japan).

### Observation of silk foothold construction behaviours

Foothold construction behaviours were observed through a transparent plastic board in videos recorded using the Stylus Tough digital camera (Olympus Co.).

### Observation of cross-sections of the passage of fibroin brins

Cross-sections of the passage of fibroin brins and adhesive, from the silk gland (or ASG) to the spigot, were prepared as described below and examined using an all-in-one fluorescence microscope (BZ-X700; Keyence Co., Japan). After anesthetising the final instar larva using CO_2_ gas, a continuous passage from the silk gland to the spinneret was carefully isolated by dissection in 0.1-M phosphate buffer. The isolated passage was immediately placed in a fixative solution [1% glutaraldehyde + 1% paraformaldehyde/0.1-M phosphate buffer (pH 7.4)] for fixing at 0 °C for 1.5 h. After fixation, samples were rinsed with the buffer and stored at 4 °C overnight. Post-fixation was conducted with a mixture of 1% osmium tetra oxide and 0.125% ruthenium tetra oxide solution at 0 °C for 1.5 h. The samples were then dehydrated using a rising ethanol series from 70 to 100% and placed into a substituting agent, n-butyl glycidyl ether (QY-1, Nisshin EM Co., Japan). Finally, the sample was embedded in an epoxy resin (Quetol-812; Nisshin EM Co., Japan), which was polymerised at 60 °C. The resultant embedded block was trimmed to expose the cross-sectional surface of the ASG, and then sections were cut from the ASG to the spigot at thicknesses of ~ 1 μm using an ultramicrotome (LKB 2088 Ultratome V; LKB, Sweden) with a glass knife. The ultrathin sections were stained with 1% toluidine blue solution containing 1% borax and then observed under a BZ-X700 optical microscope.

### 3D structural construction of the spinneret

3D X-ray microscope CT observations of the spinneret were performed using a nano3DX (Rigaku Co., Japan). The spinneret, which had been dissected from a final instar of *E. variegata* bagworm larvae, was gently washed with distilled water and then dried overnight under room conditions (25 °C and 50% relative humidity). The dried sample was fixed in an upright position on the sample stage and primarily measured using Cr characteristic X-ray radiation (5.4 keV) produced from a high-brilliance X-ray generator (MM007; Rigaku Co., Japan) with 35 kV and 25 mA, which is suitable for visualising the fine structures of organs because of the appropriate X-ray absorption contrast. The field of view in the high-resolution 2D X-ray sCMOS image sensor camera (XSight Micron LC; Rigaku Innovative Technologies Europe s.r.o., Czech Republic) was 0.6 × 0.6 mm^2^ and the effective pixel size was set to 0.3 μm. The sample was rotated by 180°and 600 projection images were captured in 900 s. Using an FDK algorithm^30^, 2000 X-ray tomograms with a thickness interval of 0.3 μm were reconstructed.

## Supplementary Information

Supplementary Information 1.

Supplementary Video S1.

Supplementary Video S2.

Supplementary Video S3.

Supplementary Video S4.

## Data Availability

Data supporting the findings of this study are available from the corresponding authors on reasonable request.

## References

[1] Epstein ME (1996). Revision and Phylogeny of the Limacodid-group Families, with Evolutionary Studies on Slug Caterpillars (Lepidoptera: Zygaenoidea).

[2] Sugiura S, Yamazaki K (2006). The role of silk threads as lifelines for caterpillars: Pattern and significance of lifeline-climbing behaviour. Ecol. Entomol..

[3] Rogóż M, Zeng H, Xuan C, Wiersma DS, Wasylczyk P (2016). Light-driven soft robot mimics caterpillar locomotion in natural scale. Adv. Opt. Mater..

[4] Brackenbury J (1997). Caterpillar kinematics. Nature.

[5] Brackenbury J (1999). Fast locomotion in caterpillars. J. Insect Physiol..

[6] Lin HT, Trimmer B (2010). Caterpillars us the substrate as their external skeleton. Commun. Integr. Biol..

[7] Griethuijsen LI, Trimmer BA (2014). Locomotion in caterpillars. Biol. Rev..

[8] Wang K, Wang W, Zhang H (2013). The mechanical properties of a wall-climbing caterpillar robot: Analysis and experiment. Int. J. Adv. Robot. Syst..

[9] Calisti M, Picardi G, Laschi C (2017). Fundamentals of soft robot locomotion. J. R. Soc. Interface.

[10] Hu W, Lum GZ, Mastrangeli M, Sitti M (2018). Small-scale soft-bodied robot with multimodal locomotion. Nature.

[11] Umedachi T, Kano T, Ishiguro A, Trimmer BA (2016). Gait control in a soft robot by sensing interactions with the environment using self-deformation. R. Soc. Open Sci..

[12] Roden DB (1993). Laddering: Climbing behavior of the Gypsy moth (Lepidoptera: Lymantriidae). Ann. Entomol. Soc. Am..

[13] Rhainds M, Davis DR, Price PW (2009). Bionomics of bagworms (Lepidoptera: Psychdae). Annu. Rev. Entomol..

[14] Yoshioka T, Tsubota T, Tashiro K, Jouraku A, Kameda T (2019). A study of the extraordinarily strong and tough silk produced by bagworms. Nat. Commun..

[15] Howard, L. O. & Chittenden, F. H. The bagworm (Thyridopteryx ephemeraeformis Haw.). In *Circular (United States. Bureau of Entomology)* Vol. 97, 1–10 (1908).

[16] Fuller C (1913). The wattle bagworm (Chalioides junodi Heylaerts). Bull. S. Afr. Dep. Agric..

[17] Bell JR, Bohan DA, Shaw EM, Weyman GS (2005). Ballooning dispersal using silk: World fauna, phylogenies, genetics and models. Bull. Entomol. Res..

[18] Rhainds M, Gries G, Ho CT, Chew PS (2002). Dispersal by bagworm larvae, Metisa plana: Effects of population density, larval sex, and host plant attributes. Ecol. Entomol..

[19] Wolff JO (2017). Strength of silk attachment to *Ilex chinensis* leaves in the tea bagworm Eumeta minuscula (Lepidoptera, Psychidae). J. R. Soc. Interface.

[20] Sahni V, Harris J, Blackledge TA, Dhinojwala A (2012). Cobweb-weaving spiders produce different attachment discs for locomotion and prey capture. Nat. Commun..

[21] Tsubota T (2020). Transcriptomic analysis of the bagworm moth silk gland reveals a number of silk genes conserved within Lepidoptera. Insect Sci..

[22] Bello B, Horard B, Couble P (1994). The selective expression of silk-protein encoding genes in Bombyx mori silk gland. Bulletin de l’Institut Pasteru.

[23] Asakura T (2007). Some observations on the structure and function of the spinning apparatus in the silkworm *Bombyx mori*. Biomacromol.

[24] Freddi G, Gotoh Y, Mori T, Tsutsui I, Tsukada M (1994). Chemical structure and physical properties of Antheraea assama silk. J. Appl. Polym. Sci..

[25] Chen F, Porter D, Vollrath F (2012). Structure and physical properties of silkworm cocoons. J. R. Soc. Interface.

[26] Lucas F, Shaw JTB, Smith SG (1960). Comparative studies of fibroins I. The amino acid composition of various fibroins and its significance in relation to their crystal structure and taxonomy. J. Mol. Biol..

[27] Craig CL, Hsu M, Kaplan D, Pierce NE (1999). A comparison of the composition of silk proteins produced by spiders and insects. Int. J. Biol. Macromol..

[28] Sutherland TD, Young JH, Weisman S, Hayashi CY, Merritt DJ (2010). Insect silk: One name, many materials. Annu. Rev. Entomol..

[29] Craig CL (1997). Evolution of arthropod silks. Annu. Rev. Entomol..

[30] Feldkamp LA, Davis LC, Kress JW (1984). Practical cone-beam algorithm. J. Opt. Soc. Am. A.

