# Supplementary material for: A study of ladder-like silk foothold for the locomotion of bagworms
Source: Sci Rep. 2021 Aug 17;11:16657. doi: 10.1038/s41598-021-95809-7 (PMC8370998; doi:10.1038/s41598-021-95809-7)
Supplement: Supplementary file 1 — Supplementary Information 1. [file 41598_2021_95809_MOESM1_ESM.pdf]

Supplementary information PDF

A Study of Ladder-like Silk Foothold for the Locomotion of Bagworms

Taiyo YOSHIOKA*, Fumiko YUKUHIRO,
Tsunenori KAMEDA*

Silk Materials Research Group, National Agriculture and Food Research Organization (NARO), 1-2 Owashi, Tsukuba, Ibaraki 305-8634, Japan

***Authors for correspondence**

Taiyo YOSHIOKA and Tsunenori KAMEDA

Silk Materials Research Group, National Agriculture and Food Research Organization (NARO), 1-2 Owashi, Tsukuba, Ibaraki 305-8634, Japan

Tel: +81-29-838-6172 and +81-29-838-6213

E-mails: yoshiokat@affrc.go.jp and kamedat@affrc.go.jp

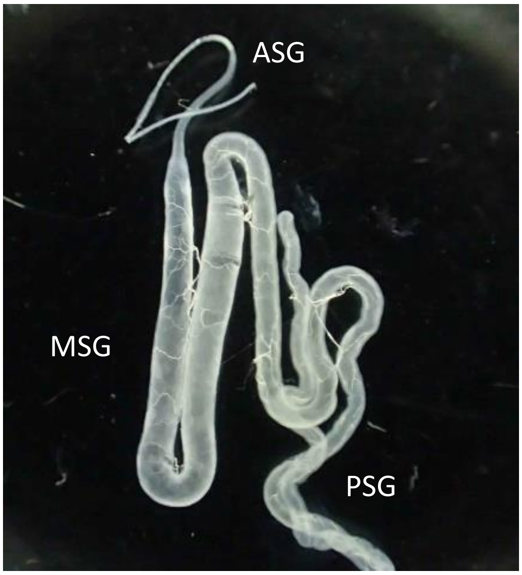

Supplementary Figure S1. Optical micrograph of one side of a pair of silk glands.

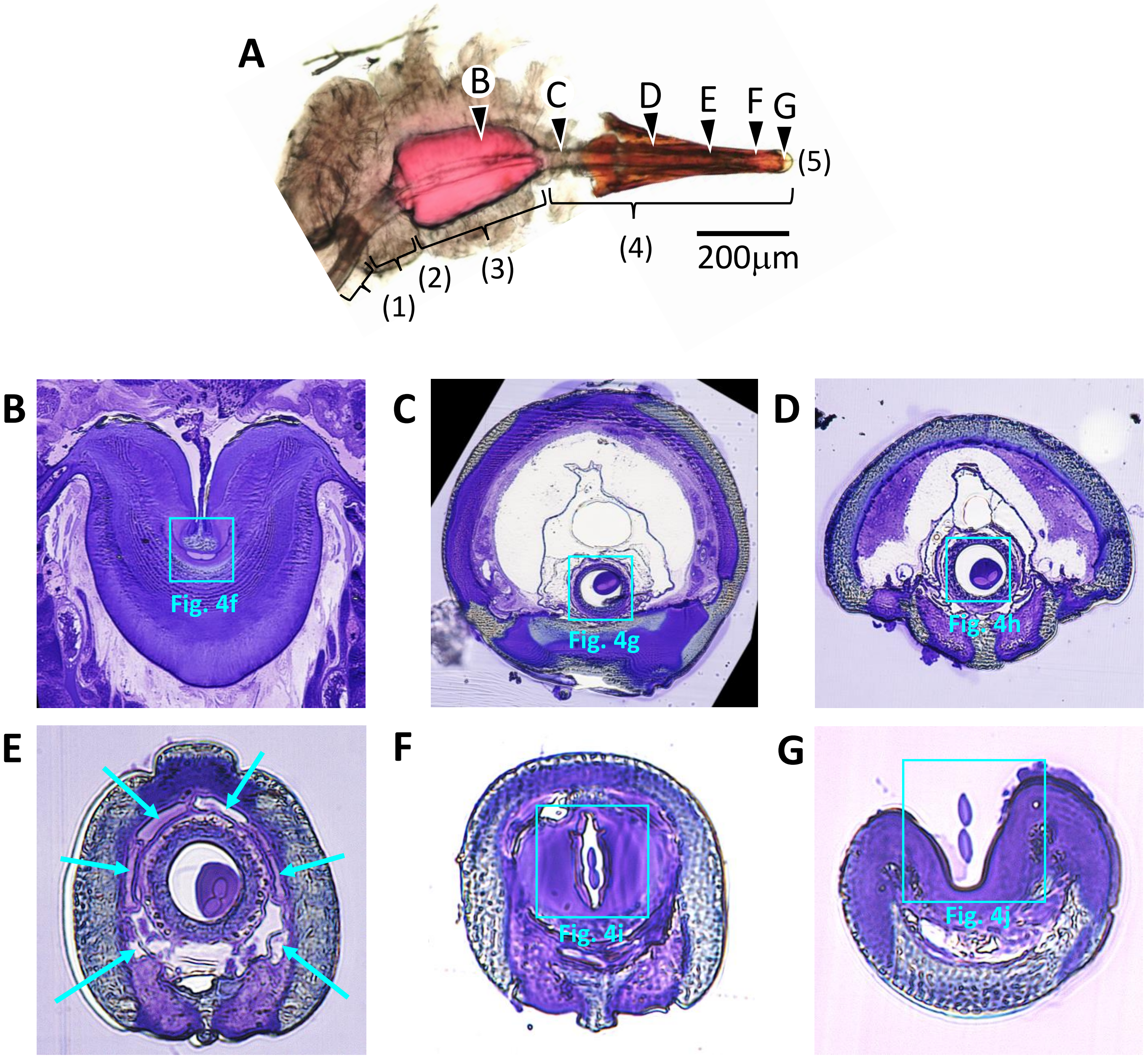

Supplementary Figure S2. Structures of the passages of fibroin brins and adhesive. (**A**) An optical microscope image of the passage (identical to Fig. 4b) including the (1) end of the anterior silk glands, (2) common tube, (3) silk press, (4) spinning tube and (5) spigot. (**B**–**G**) Optical microscope images of the respective cross-sections obtained from positions (**B**–**G**) in (**A**). The images (**B**–**D**)**,** (**F**) and (**G**) correspond to the respective unmagnified views of the images f–j in Fig. 4. Three pairs of plural adhesive ducts are indicated by arrows in (**E**).

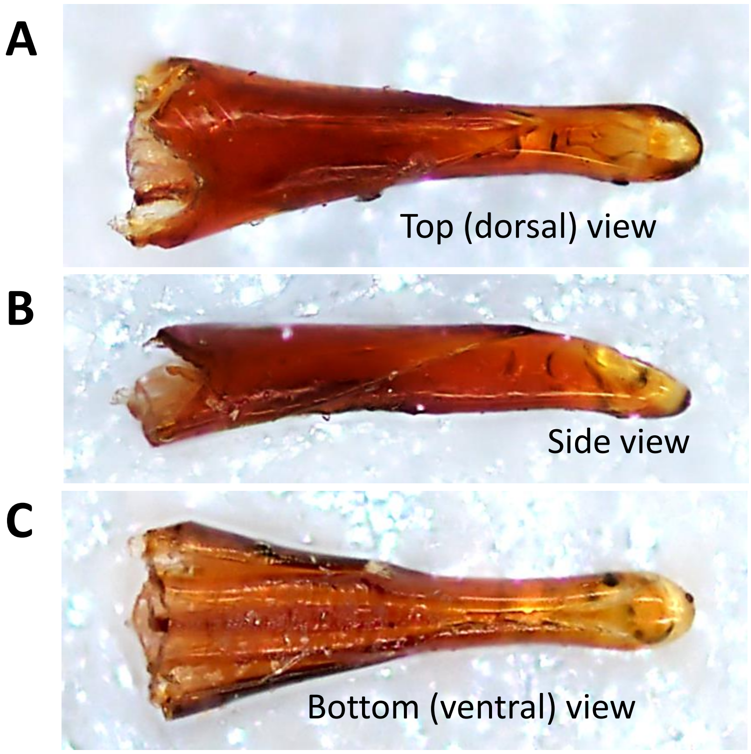

Supplementary Figure S3. The exterior shape of the spinning tube. Optical microscope images of the spinning tube: (**A**) dorsal view, (**B**) side view and (**C**) ventral view.

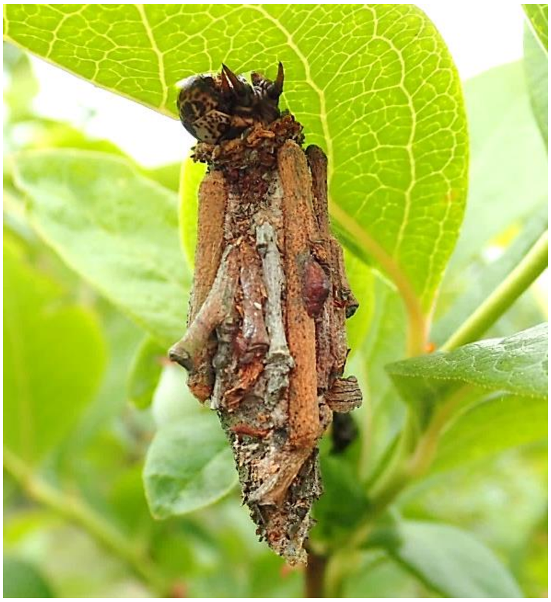

Supplementary Figure S4. A bagworm (*Eumeta minuscula*) walking on the bottom side of a leaf.

Supplementary Movie S1.

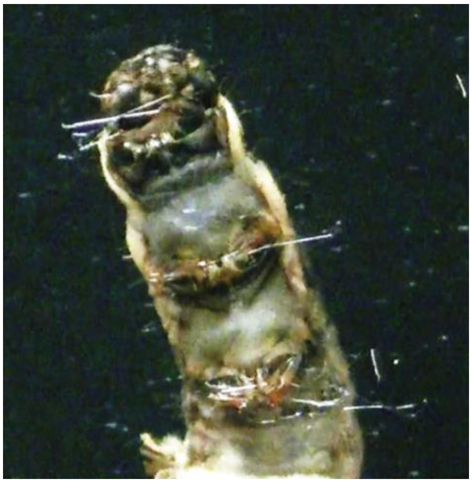

Walking behaviour of bagworms.

Supplementary Movie S2.

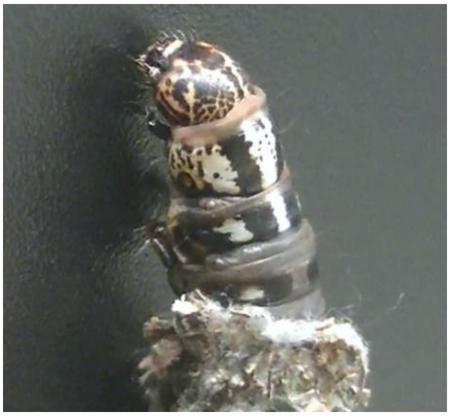

An *Eumeta minuscula* bagworm
climbing on a slippery wall.

Supplementary Movie S3.

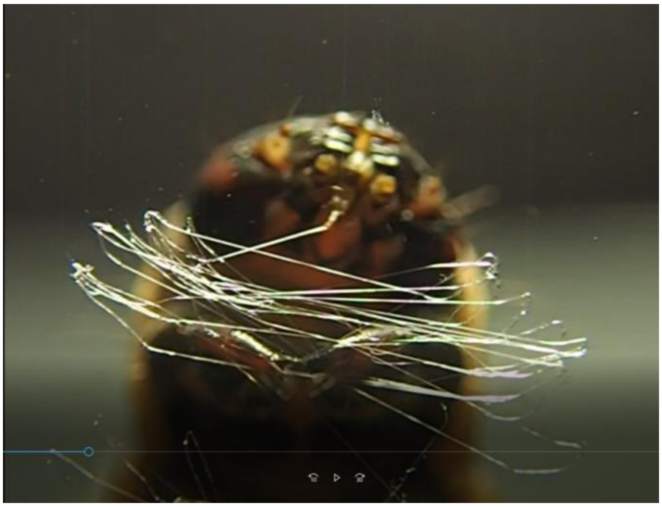

Construction behaviour of silk foothold.

Supplementary Movie S4.

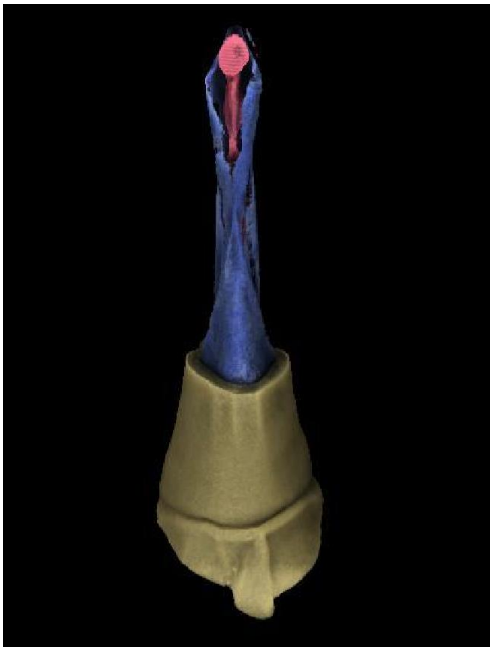

3D structure of spinneret revealed by X-ray CT nano3DX (Rigaku Co., Japan).
